# Evaluating shelf life and anti-browning of shrimp by chitosan-coated nanoliposome loaded with licorice root extract

**DOI:** 10.1016/j.fochx.2024.101532

**Published:** 2024-06-04

**Authors:** Masume Kamali, Bahare Shabanpour, Parastoo Pourashouri, Moazameh Kordjazi

**Affiliations:** Department of Seafood Processing, Faculty of Fisheries and Environmental Science, Gorgan University of Agriculture Sciences and Natural Resources, Gorgan, Iran

**Keywords:** Melanosis, White-leg shrimp, *Glycyrrhiza glabra*, Encapsulation, Ice storage

## Abstract

Chitosan coating containing nanoliposomes loaded with licorice root extract was prepared to investigate shrimp's shelf life and anti-browning function during 20 days of ice storage. 1% licorice root hydroethanolic extract (LHE) was encapsulated in nanoliposomes or coated with chitosan, and then the shrimp were immersed in coating solutions. LHE treatment had the lowest browning indices (5 and 1.02), TBA (0.32 mg MDA/kg), and FFA (0.01%). Chitosan-coated LHE treatment (Ch-LHE) showed the best performance for TVN, microbial counts, and discoloration. PV, WHC, and cook loss in the treatment with LHE nanoliposome coated with chitosan (Ch-N-LHE) were measured at acceptable levels of 0.53 meq/kg, 86.12%, and 15.06%, respectively. Experiments showed that pure or encapsulated LHE is an effective method for increasing the quality and preventing the browning of shrimp. Additionally, due to its cost-effectiveness and health benefits, it can be an effective natural substitute for sodium metabisulfite at the global export level.

## Highlights

• Licorice root hydroethanolic extract (LHE) could decrease the browning of shrimp.
• LHE increased shelf life and improved shrimp chemical properties (TBA and FFA).
• Chitosan-coated LHE served best in TVN, microbial counts, and shrimp color change.
• LHE loaded in nanoliposomes showed significant effects on physical characteristics.
• LHE is a promising non-sulfite anti-browning agent from natural sources.

## 1 Introduction

White-leg shrimp is one of the world's most famous and widely consumed species due to its high economic and nutritional value. Despite its high nutritional value and the increasing trend of global shrimp production, microbial spoilage and melanosis limit its consumption (Liang, Lin, Chen, & Sun, 2022).

Melanosis in shrimp is initiated by an enzymatic reaction that even ice or refrigeration storage cannot prevent. In this reaction, polyphenol oxidase (PPO) is activated, causing the phenolic compounds in shrimp to oxidize into dark-colored compounds. Non-enzymatic polymerization then converts the quinones into high molecular weight dark pigments called melanin, found under the carapace of the cephalothorax of the shrimp (Gokoglu, Gumus, Ceylan, & Gokoglu, 2022). This browning process reduces the shrimp's market value and results in significant financial losses.

Sulfite compounds are often used to overcome or reduce browning in shrimp and other crustaceans. However, these chemical compounds can cause allergic reactions, breathing problems, and, in rare cases, anaphylaxis in some people (Isfran, Chacon, Alves, Monteiro, & Ayala Valencia, 2023).

So far, chemicals such as acids and thermal processing methods have been used, but these have led to reduced quality and denatured shrimp (J. Wang et al., 2018). As a result, there is an urgent need to discover new and safer alternatives. Natural additives such as plant extracts can be a suitable solution and a better choice. Several plant and algal extracts have shown promising anti-melanosis effects on various crustaceans. Pomegranate peel extract (Yuan, Lv, Tang, Zhang, & Sun, 2016), chamoang leaf extract (Shiekh, Benjakul, & Sae-Leaw, 2019), hyssop plant essential oil (Mehraie, Khanzadi, Hashemi, & Azizzadeh, 2023), and green algae polysaccharide Ulva (Kamali, Shabanpour, Pourashouri, & Kordjazi, 2023) have been successfully used to delay microbiological and chemical spoilage and to inhibit melanosis.

A useful and readily available natural substance is the licorice plant (*Glycyrrhiza glabra*), which is native to tropical regions and the Middle East and is usually found in southern Iran and Russia. Some experiments have shown that licorice acts as a tyrosinase inhibitor and contains active chemicals such as glabridin and antioxidants that can help reduce the browning of the mushroom (Pan et al., 2023).

Directly using natural extracts and additives in food products often leads to high release rates and reduced packaging activity during storage. Therefore, nanoencapsulation emerges as an efficient method to enhance the effectiveness, physical stability, and biological activity of natural extracts. Nanoliposomes serve as flexible carriers capable of simultaneously transporting hydrophobic and hydrophilic substances within a bilayer membrane or inside a vesicle. This characteristic allows for better control of release and more effective delivery of encapsulated compounds to target cells (Mehdizadeh, Shahidi, Shariatifar, Shiran, & Ghorbani-HasanSaraei, 2021).

To enhance the structural, physical, and mechanical properties for controlling the release of encapsulated compounds, a novel approach involving the surface coating of nanoliposomes with biopolymers such as chitosan is proposed. Chitosan, known for its film-forming properties, has been increasingly utilized in recent years due to its biocompatibility, ability to extend shelf life, and quality of marine products (Mehraie et al., 2023).

Considering the anti-tyrosinase property of the licorice plant and the availability of this natural substance, there is a perceived necessity to utilize it with a new microencapsulation method to enhance effectiveness in maintaining the quality, transportation chain, and export of shrimp. In the present study, a hydroalcoholic extract of licorice root was produced and loaded at a concentration of 1% into nanoliposomes, which were subsequently coated with chitosan. Subsequently, the impact of various coatings containing licorice hydroalcoholic extract (LHE) was investigated on the physicochemical, microbial, textural, sensory, and anti-browning characteristics of white-leg shrimp during ice storage.

## 2 Materials and methods

### 2.1 Materials

Lecithin (Soybean phospholipid; PubChem CID: 5287971), Chitosan (C56H103N9O39) (PubChem CID: 71853), Thiobarbituric acid; 4,6(1H,5H)-Pyrimidinedione, dihydro-2-thioxo-; (PubChem CID: 2723628), and Trichloroacetic acid (PubChem CID: 6421) were purchased from Sigma-Aldrich (St. Louis, Missouri, USA). Ammonium sulfate (PubChem CID: 6097028); ethanol (PubChem CID: 702), Trichloroform (PubChem CID: 6212), and GranuCult™ (Plate Count agar (PCA)) were purchased from Merck (Darmstadt, Germany). Sodium metabisulfite (PubChem CID: 656671), and phenolphthalein (PubChem CID: 4764) were obtained from Tetra-Chem (Ingersoll, Canada). All the chemical reagents used in this study were of analytical grade.

### 2.2 Licorice root extract preparation

Licorice root (*Glycyrrhiza glabra*) was procured from the herbal medicine market in Gorgan, Iran. The licorice root powder was dissolved in ethanol/water (70:30 *v*/v) at a ratio of 1:10 (g/mL) and agitated at room temperature (200 Vib/min) for 24 h. The resulting extract was filtered, and the solvent was then evaporated at 50 °C using a rotary evaporator (Buchi, Switzerland). The dried extract was subsequently stored at −20 °C.

### 2.3 Shrimp collection and preparation

Freshly caught white-leg shrimp (*Litopenaeus vannamei*), with a size range of 60 to 65 shrimp per kilogram, was sourced from Gomishan shrimp farm in Gorgan, Iran. Upon capture, the shrimp were immediately placed in ice at a ratio of 1:2 (shrimp to ice, *w*/w) and transported to the laboratory within 1 h. Upon arrival, the shrimp underwent a gentle wash in ice water (maintained at 1–3 °C) and were subsequently stored in ice until further use, with a total duration of <1 h.

### 2.4 Coatings preparation

#### 2.4.1 Nanoliposome preparation

A solution containing 5% (*w*/w) lecithin was prepared by mixing 2 g of lecithin with 38 mL of deionized water. The mixture was blended at room temperature for 4 h. Subsequently, the suspension underwent sonication for 180 s at 40 kHz and 40% power (1 s on and 1 s off) using a Topsonics Sonicator in Iran, resulting in a colloidal suspension. The nanoliposomes were then stored in sterile bottles at 4 °C in the dark, as per the methodology outlined by Kamali et al. (2023).

#### 2.4.2 Nanoliposome/chitosan mixture and trial solutions preparation

1% chitosan solution was prepared in acetic acid (1% *v*/v) and stirred for 2 h. Subsequently, 1% LHE solution was created by dissolving 1 g of LHE in 100 mL of deionized water. For the formation of LHE nanoliposome coating (N-LHE), 1 g of LHE was dissolved in 100 mL of lecithin solution and subjected to sonication. To obtain chitosan-coated LHE (Ch-LHE), 1 g of LHE was added to 100 mL of chitosan solution. Next, 20 mL of LHE-containing nanoliposomes were mixed with 80 mL of chitosan solution (*v*/v) for 48 h in the dark to form the chitosan coating of LHE nanoliposomes (Ch-N-LHE), as outlined by Kamali et al. (2023).

### 2.5 Immersion of shrimp in trial solutions

The shrimps were randomly divided into six groups: control (uncovered), sodium metabisulfite (SMS), LHE coating, LHE nanoliposome (N-LHE), LHE chitosan coating (Ch-LHE), and LHE nanoliposome chitosan coating (Ch-N-LHE). Shrimps in the different LHE groups were immersed in the respective solutions at a shrimp-to-solution ratio of 1:2 (*w*/*v*) at 4 °C for 30 min. After immersion, the shrimps were drained at room temperature for 3 min. Additionally, another portion of the shrimp was treated with 1.25% SMS solution dissolved in deionized water at a shrimp-to-solution ratio of 1:2 (w/v) for 1 min at 4 °C. Following treatment, the samples of each group were sealed in plastic bags and placed in ice with a shrimp-to-ice ratio of 1:2 (*w*/w) in insulated boxes, and then stored in a refrigerator. To maintain the shrimp-to-ice ratio, any melted ice was removed and replaced with an equal volume of ice daily.

### 2.6 Physical and biochemical experiments

The shrimps were weighed on days 0, 4, 8, 12, 16, and 20, respectively, to monitor weight changes over time. Weight loss (%) was calculated by determining the percentage of initial weight lost at the beginning of storage, following the methodology outlined by Kamali et al. (2023). pH measurement was conducted using a digital pH meter (Metrohm, Switzerland, model 713) according to the ISO 2917 (1999) standard. To assess cooking loss, the drained shrimps were steam-cooked for 5 min at 99 °C. Cooking loss (%) was determined by comparing the weight loss percentage before and after cooking the peeled shrimp.Water holding capacity (WHC) was determined following the method described by Li et al. (2022).

### 2.7 Chemical tests

Following the AOAC Official Method 965.33 (2016) using a Soxhlet apparatus, 5 g of the sample was combined with 2 g of magnesium oxide in a thermal flask containing 300 mL of distilled water. In the receiving vessel, a few droplets of phenolphthalein indicator were added to 25 mL of 2% boric acid solution. The evaporator was connected to both vessels (heating and receiving), and the water temperature was maintained. After 3 min, distillation was ceased. The content of the receiving flask was titrated to the endpoint using very dilute 0.05 M sulfuric acid (H_2_SO_4_). The determination of total volatile nitrogen (TVN) was conducted as described below:

(1) $$\mathrm{TVN}\;\left(\mathrm{mg}/100\;g\right)=\left(V\times N\times 100\times 14\right)/W$$

where: V = volume (mL) of H_2_SO_4_ used for the sample. N = normality of H_2_SO_4_ W = weight of sample in g.

The TBA test was performed based on the method of Zhang, Yan, Su, and Chen (2020). The sample (0.5 g) was combined with a TBA reagent containing 2.5 mL of 0.375% TBA, 15% TCA, and 0.25 N HCl. The solution was boiled in a water bath for 10 min, then cooled and centrifuged for 10 min at 5000*g* and 25 °C. The absorbance of the supernatant was determined at 532 nm. TBA was measured in the sample as MDA equivalents (mg MDA/kg) using a standard curve created with malondialdehyde at concentrations ranging from 0 to 10 ppm.

The peroxide value (PV) was determined in accordance with ISO 3960 (2017). Samples (3 g) were weighed into a glass-stoppered Erlenmeyer flask. The sample was heated for 3 min at 60 °C in a water bath to melt the fat. The container was thoroughly shaken for 3 min with 30 mL of an acetic acid-chloroform solution (3:2 *v*/v) to dissolve the fat. Shrimp pieces were removed using Whatman No. 1 filter paper during the filtration process. After adding saturated potassium iodide solution (0.5 mL) to filtration, the reaction continued by adding starch solution as an indicator. Titration was performed with a standard sodium thiosulfate solution. The peroxide value, reported as milliequivalents of peroxide per kilogram of sample, was calculated using the following equation:

(2) $$\mathrm{PV}\;\left(\mathrm{meq}/\mathrm{kg}\right)=\left[\left(S\times N\right)/W\right]\times 100$$

where: S = volume of used sodium thiosulfate (mL), N = normality of sodium thiosulfate solution (*N* = 0.01), and W = sample weight (g).

The value of free fatty acids (FFA) was determined using the official analytical method ISO 660 (2009). A homogenizer was used to dissolve the sample (5 g) in 30 mL of chloroform for 1 min at 10,000 rpm. Shrimp pieces were removed using Whatman No. 1 filter paper. After adding five drops of 1% ethanolic phenolphthalein as an indicator to the filtrate, titration was performed with 0.01 N ethanolic potassium hydroxide solution. The FFA value was then calculated using the following equation:

(3) $$\mathrm{FFA}\;\left(\%\right)=\left(\mathrm{mL}\;\text{titration}\times \text{Normality of}\;\mathrm{KOH}\times 28.2\right)/g\;\text{sample}$$

### 2.8 Microbial tests

One gram of shrimp meat was mixed with 9 mL of 0.85% sterile saline solution. The mixture was then homogenized for 2 min at 250 g. The homogenized solution was logarithmically diluted in a sterile physiological saline solution. One milliliter of each dilution was inoculated onto plate count agar (PCA) culture medium. The cultured samples were incubated at 35 °C for two days to determine the total viable count (TVC) and at 4 °C for seven days to count the number of psychrophilic bacteria (PBC), in accordance with ISO 4833-2 (2013). After the incubation period, the number of colonies was counted.

### 2.9 Texture profile analysis

A texture analyzer LFRA-4500 (Brookfield Engineering Laboratories, Inc., Middleboro, MA) was used to evaluate the texture (hardness, gumminess, springiness, chewiness, and cohesiveness) of shrimp muscle samples based on the official analytical method ISO 11036 (2020). The texture profile analysis (TPA) was performed at room temperature under the following conditions: constant test speed, 1.0 mm/s; deformation of the whole shrimp meat sample, 50%; hold time between cycles, 3 s; and trigger force, 0.05 N. Texture analysis parameters were measured using Texture Pro-Lite V1.0 software from the force-time curve generated from each sample.

### 2.10 Sensory evaluation

The sensory evaluation performed in this study was conducted with the approval and consent of the participants, based on the method of ISO 11136 (2014). Twelve students from the Department of Fisheries at Gorgan University of Agricultural Sciences and Natural Resources (6 men and 6 women, aged 25 to 35) voluntarily participated, ensuring the safety of the samples under evaluation. Participants were required to be regular users of these types of products. The panelists rated the appearance, overall acceptability, smell, texture, and color of the different shrimp samples using organoleptic evaluation (sight, smell, and touch). Each parameter was rated separately on a descriptive hedonic scale from 1 to 9. Sensory evaluation was conducted under standard conditions in a laboratory with at least 50 square meters of space, a circulating air system with three ventilations per hour, and without noise and odor pollution. The temperature was maintained at 20–22 °C, with relative humidity at 50–60%. The illumination was 650–750 lx, provided by fluorescent lamps with a color temperature of 5500–6000 Kelvin. The room height was 4 m, and the color of the test area and equipment was neutral (white or light gray).

### 2.11 Shrimp shell color measurement

Shrimp color was measured using an automatic colorimeter (Hunter Lab, Lovibond, CAM-System 500, UK) according to ISO 11037 (2011). The L* value (lightness) ranges from 0 (dark) to 100 (white), indicating brightness. The a* value indicates redness, ranging from negative (green) to positive (red), and the b* value indicates yellowness, ranging from negative (blue) to positive (yellow). Shrimp shell color was assessed in three parts: cephalothorax, body, and tail. The following equations were used to determine chroma (C*), hue (H*), browning index (BI), and the total color difference (ΔE), which indicates the amount of color difference between shrimp before and after storage:

(4) $$C^{*}={\left({a^{*}}^{2}+b^{*2}\right)}^{1/2}$$

(5) $$H^{*}=\tan^{-1}\;\left[b^{*}/a^{*}\right]$$

(6) $$\mathrm{BI}=\frac{100\;}{0.17}\;\left(\frac{a^{*}+1.75\;L^{*}}{\;5.645\;L^{*}+a^{*}-0.012\;b^{*}}-0.31\right)$$

(7) $$\mathrm{\Delta E}={\left[{\left(L^{*}-{L^{*}}_{0}\right)}^{2}+{\left(a^{*}-{a^{*}}_{0}\right)}^{2}+{\left(b^{*}-{b^{*}}_{0}\right)}^{2}\right]}^{1/2}$$

The values of the color parameters L *, a *, and b * at the beginning of storage are denoted as L *_0_, a *_0_, and b *_0_, respectively.

### 2.12 Browning function assessment

The white-leg shrimp browning function was assessed by twelve participants using a ten-point ranking model based on the International Organization for Standardization standard ISO 8586-1 (2023). Panel members were asked to assign a browning score ranging from 0 to 10 to the shrimp. A score of 0 indicated no browning function, while a score of 2 indicated partial browning affecting up to 20% of the shrimp surface. A score of 4 represented medium browning (affecting 20% to 40% of the shrimp surface), 6 indicated considerable browning (affecting 40% to 60% of the shrimp surface), 8 indicated heavy browning (affecting 60% to 80% of the shrimp surface), and 10 indicated very heavy browning (affecting >80% up to the total shrimp surface). For the evaluation of the browning function, sampling was conducted once every four days over a period of 20 days for each treatment.

### 2.13 Statistical analysis

Each measurement was repeated three times, and statistical data analysis was conducted using SPSS version 16.0. Statistical analyses were performed using one-way ANOVA (Duncan and LSD post-test analysis). The data were presented as mean ± standard deviation, and statistical significance was considered at *p* < 0.05 for the difference between means.

## 3 Results and discussion

### 3.1 Physical analysis

#### 3.1.1 Loss of weight

Weight loss of products is one of the physical aspects that can affect their texture and sensory quality. The sample's weight loss increased significantly over time (p < 0.05) (Table 2). On the last day of storage, the lowest and highest weight loss were observed in N-LHE and SMS treatments with values of 13.83 and 43.29%, respectively. In this study, it can be inferred that LHE nanoliposome coating is an acceptable option to reduce shrimp moisture loss. It is possible that the plant extract in the nanoliposome coating acted as a semi-permeable layer around the surface, thereby preventing moisture loss in the samples. In this context, nanoliposomes have been widely developed as carriers of bioactive substances for two main reasons they can overcome temperature extremes and extreme pH, and they can improve the controlled delivery of bioactive compounds to target cells. This result is consistent with previous studies. For instance, Q. Wang, Lei, Ma, Yuan, and Sun (2018) reported that chitosan-carvacrol coating significantly reduced the moisture loss of shrimp during 10 days of ice storage due to its water vapor barrier properties. Similarly, Farajzadeh, Motamedzadegan, Shahidi, and Hamzeh (2016) stated that chitosan-gelatin coating effectively protected chilled Pacific white shrimp against water loss during 14 days of refrigerated storage, with a loss rate of 5.09%.

#### 3.1.2 PH

The pH changes of white-leg shrimp with different treatments during ice storage are presented in Table 2. According to a report by Çolakoğlu, Ormancı, and Altın (2006), a pH of 7.7 or less for shrimp indicates excellent quality, 7.70–7.95 indicates acceptable quality, and 7.95 or higher represents poor quality. The initial pH of fresh white-leg shrimp on day zero was 6.7. As storage time increased, the pH of all shrimp increased significantly (*p* < 0.05). The increase in pH during storage is typically attributed to the process of autolysis of endogenous enzymes, production of alkaline substances such as trimethylamine and ammonia, and the increased activity of microbial enzymes (Anvar, Nateghi, Shariatifar, & Mousavi, 2023). The increase in pH was significantly inhibited in Ch-LHE and Ch-N-LHE-treated shrimp (7.24 and 7.31, respectively). Therefore, this decrease may be attributed to the ability of chitosan and nanoliposomes to control or prevent the growth of bacteria, thereby preventing protein hydrolysis and contributing to increased durability and decreased pH of shrimp samples. This research is analogous to the study conducted by Homayonpour, Jalali, Shariatifar, and Amanlou (2021), which found that storing fish at 4 °C with *Cuminum cyminum* L. essential oil nano had a more beneficial effect on pH compared to free essential oil. In addition, in a similar study, *Salmo trutta* coated with chitosan and *zataria multiflora* essential oil exhibited the lowest pH value (6.87) during 16 days of storage (Anvar et al., 2023). These findings suggest that LHE coating can delay the degradation of shrimp quality and consequently reduce spoilage or decomposition

#### 3.1.3 Cook loss

Cooking loss refers to the loss of soluble and liquid substances during the cooking process. The measured values for cooking loss of the samples are presented in Table 2. While all specimens exhibited a slight increase in cooking loss during the middle of the storage period, by the end of storage, all treatments showed a decrease in cooking loss. The lowest cooking loss was observed in the control and Ch-N-LHE treatments, with 14.62% and 15.06%, respectively, while the highest cooking loss was observed in the N-LHE treatment, with 24.15%. This difference may be attributed to the bilayer structure of nanoliposomes. When nanoliposomes are subjected to high temperatures, the liposome membrane undergoes a transition into a gel state, leading to the disappearance of the bilayer structure and an increase in membrane permeability. Subsequently, the gel state transforms into a liquid state. As a result, the liquid released from the nanoliposome membrane, along with soluble substances and lost liquid during meat heating, contributes to an increase in cooking loss. Similarly, in a study by Wang, Tang, et al. (2018), cooking loss ranging from 17% to 36% was reported for northern white shrimp (*Litopenaeus setiferus*) at treatment temperatures of 60 °C to 100 °C. Additionally, in the research conducted by Sun et al. (2023), cooking loss in the muscle of white shrimp was observed to be reduced using magnetic field-assisted immersion freezing (MF) compared to immersion freezing (IF). During the MF process, the appropriate magnetic field intensity can increase the amount of bound non-freezable water, thereby better maintaining the stability of the protein structure. With the heating process, contraction and tightening of the muscle tissue increases the internal pressure and leads to the release of water from the meat tissue. Consequently, the weight of the shrimp decreases.

#### 3.1.4 WHC

Water holding capacity (WHC) is indicative of protein degradation and shrimp meat quality. The WHC of all samples remained consistently high, fluctuating between 70% and 88% throughout the storage period (Table 2). Towards the end of the storage period, most treatments exhibited a slight decrease in WHC. The highest WHC values were observed in the Ch-N-LHE and SMS treatments, with values of 86.2% and 88.53%, respectively. In the Ch-N-LHE treatment, the chitosan or nanoliposome coatings likely create a semipermeable layer around the shrimp surface, thereby reducing moisture loss. In SMS treatment, the alkaline pH may positively influence tissue WHC. In justification of this phenomenon, it is worth noticing that the increase in muscle pH causes the dissolution of a series of proteins, hence enhancing the water binding to the protein, which results in the increment of the water storage capacity in the muscle. Martinez-Alvarez, Lopez-Caballero, Montero, and del Carmen Gomez-Guillen (2020) also reported similar WHC values (approximately 77–88%) in deep-water shrimp from the beginning to the end of frozen storage. Similarly, in the study by Sun et al. (2023), the water-holding capacity of shrimp using magnetic field (MF) immersion freezing was the highest among all frozen samples, at about 93%. It's noteworthy that both weight loss and cooking loss contribute to the WHC of the meat, as higher weight loss leads to lower WHC in the meat sample.

### 3.2 Chemical analyses

#### 3.2.1 TVN

**Fig. 1:**
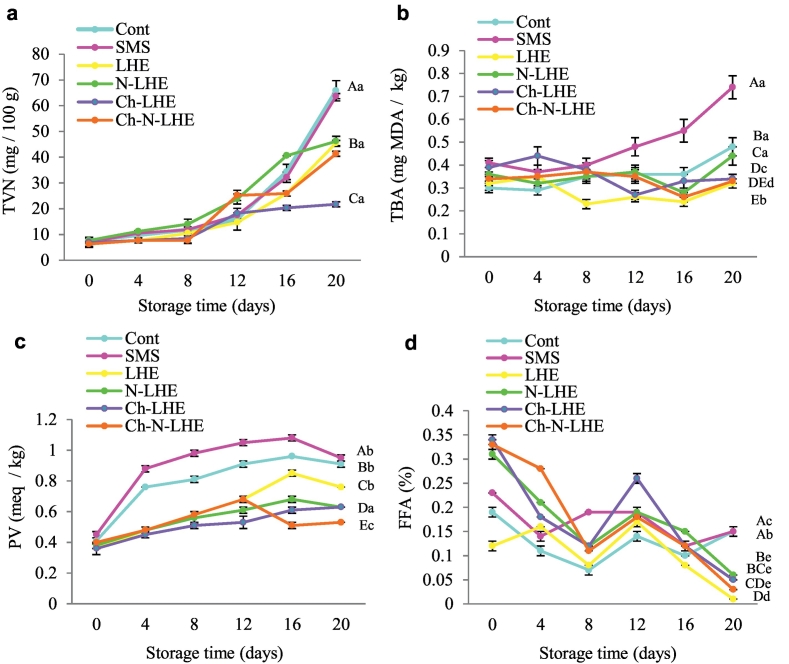
Changes in Total volatile nitrogen (TVN) (a), Thiobarbiuturic acid (TBA) (b) Peroxide value (PV) (c), and Free fatty acid (FFA) (d) (mean ± SD) of white leg shrimp with various treatments during iced storage. The bars represent the standard deviation (*n* = 3). Uppercase and lowercase letters on the bars indicate significant differences in different treatments and days, respectively (*p* < 0.05).

Total volatile base nitrogen (TVN) serves as an indicator of spoilage and is mainly attributed to the presence of ammonia produced by bacterial catabolism of nitrogen-containing compounds (Liu, Zhu, Yang, Hu, & Jiang, 2022). The maximum recommended limit (MRL) for TVN in most countries is set at 30 mg per 100 g. An increase in TVN content was observed in all samples with increasing storage time (*p* < 0.05) (Fig. 1a). TVN measurements show that Ch-LHE-coated shrimp have a shelf life of 20 days in ice storage (below 30 mg/100 g) (p < 0.05). Specifically, the TVN content in the Ch-LHE treatment was 21.7 mg N/100 g. Chitosan-based films, when used as nanocarriers of bioactive compounds, enable the development of functional packaging with antimicrobial and antioxidant properties. This system is highly desirable for ensuring the quality, safety, and shelf life of marine food products. These coatings may have helped to preserve the antibacterial properties of LHE over an extended period. Similarly, *Hyssopus officinalis* essential oil combined with chitosan, both in emulsion and nanoemulsion forms, demonstrated a positive effect in preventing the increase of shrimp TVN during 12 days of storage by inhibiting microbial activity (Mehraie et al., 2023). Also, the amount of TVN in rainbow salmon fillet coated with basil seed gum containing *Foeniculum vulgare* essential oil (free and nano) reached 15.32 mg N/100 g during 28 days of storage in the refrigerator (Sayyari, Rabani, Farahmandfar, Esmaeilzadeh Kenari, & Mousavi Nadoshan, 2021). Similarly, in a study by Anvar et al. (2023), the TVN content in minced meat nano-encapsulated with *Anethum graveolens* L. essential oil was lower than in its free form during 18 days of refrigerated storage, at 27 mg N/100 g.

#### 3.2.2 TBA

The level of TBA is used to determine the oxidation of products. At the end of storage, the TBA value of LHE-treated shrimp remained constant at 0.32 mg MDA/kg, while the Ch-LHE and Ch-N-LHE treatments showed a slight decrease, with values of 0.34 and 0.33 mg MDA/kg, respectively (Fig. 1 b). The immutability of oxidation in LHE-treated shrimp can be attributed to its radical scavenging activity, or LHE may chelate with metal oxidants present in the shrimp tissue. It is also possible that shrimp treated with LHE are more resistant to lipid oxidation than other samples, effectively slowing down lipid oxidation in shrimp tissue. Similarly, Li et al. (2022) reported that shrimp treated with phytic acid and lactic acid, and then cooked, exhibited the best inhibitory effect on lipid oxidation during 18 days of storage at 37 °C, likely due to their antioxidant activity. Another study demonstrated a decrease in TBA levels in *Litopenaeus vannamei* shrimp coated with a chitosan emulsion containing 1% *Hyssopus officinalis* essential oil during 12 days of refrigerated storage, compared to other treatments, which confirms the results of the present study (Mehraie et al., 2023). Similarly, Esmaeili et al. (2020) reported that sausages coated with a chitosan edible film containing nano-encapsulated garlic essential oil showed the best TBA results compared to the control sample over 50 days.

#### 3.2.3 PV

Peroxide values (PV) are shown in Fig. 1 c. PV of all samples increased with increasing storage time (*p* < 0.05). However, the PV of all treatments decreased on the last day of storage. The lowest PV was observed in the Ch-N-LHE treatment at 0.53 meq/kg, while the highest was in the SMS treatment at 0.95 meq/kg (*p* < 0.05). The increase in PV may be attributed to the presence of fatty acids in the shrimp tissue, which undergo oxidation during storage, leading to the formation of peroxides or hydroperoxides. Chitosan coatings have been reported as effective barriers against oxygen permeability and may act as a protective layer between the shrimp surface and the surrounding environment. Since all samples had less than ten meq/kg of fat, this falls within an acceptable limit. These results align with the findings of Liu et al. (2022), who observed that chitosan coating with *Hyssopus officinalis* essential oil nanoemulsion exhibited superior performance in preventing lipid oxidation in shrimp. Similarly, in another study, treatment with chitosan coating containing *Zataria multiflora* essential oil showed the lowest PV value of 0.78 meq/kg in *Salmo trutta* after 16 days of storage (Zomorodian, Javanshir, Shariatifar, & Rostamnia, 2023).

#### 3.2.4 FFA

Changes in FFA levels among different treatments during ice storage are depicted in Fig. 1d. The results indicate that the LHE and Ch-N-LHE treatments exhibited the lowest FFA levels, with values of 0.01% and 0.03%, respectively, over the 20-day storage period (p < 0.05). The observed decrease in FFA levels may be attributed to the inhibitory effect of the various coatings applied on the amount of lipolysis in the shrimp samples or their antioxidant properties. Additionally, the function of chitosan or nanoliposome solutions as a barrier against water evaporation could contribute to reducing the rate of fat hydrolysis. Chitosan coating and the stability of nanoliposomal phospholipids lead to less FFA release, and the rate of FFA release of nanoliposomal phospholipids is a valuable indicator for nanoliposome integrity. Mehraie et al. (2023) reported that an edible coating containing chitosan nanoemulsion significantly reduced the amount of FFA in shrimp during a 12-day storage period. Similarly, in the study by Alparslan, Baygar, Metin, Yapici, and Baygar (2019), gelatin film combined with orange peel essential oil groups effectively prevented the formation of free fatty acids in deepwater pink shrimp over a 15-day storage period. Also, a change in FFA content similar to our study was reported by Reesha, Panda, Bindu, and Varghese (2015), in tilapia with an LDPE/chitosan composite antimicrobial film during 30 days of storage period, with a recorded value of 6 mg% oleic acid.

### 3.3 Microbial analysis

#### 3.3.1 Total viable counts (TVC)

**Fig. 2:**
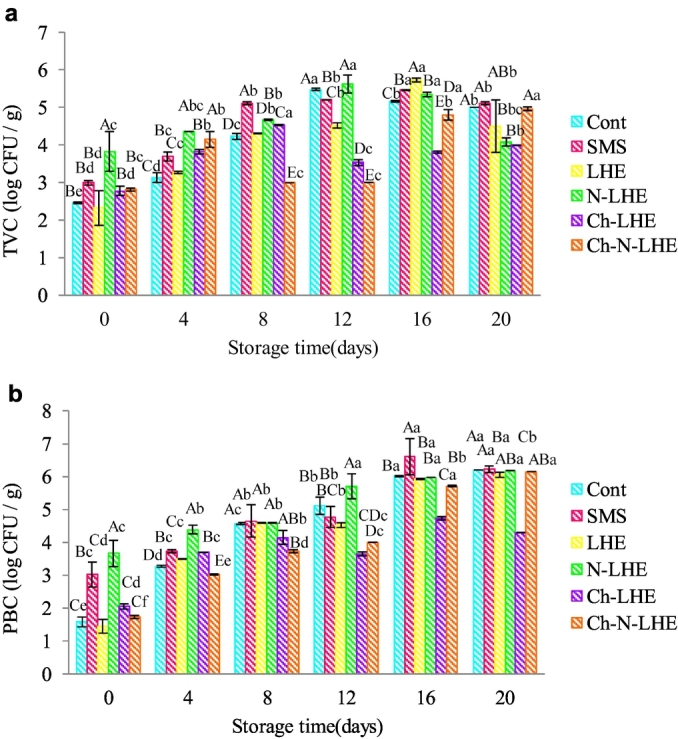
Changes in the total viable bacterial count (TVC) (a) and psychrophilic bacteria count (PBC) (b) (mean ± SD) of white leg shrimp with various treatments during ice storage. Uppercase and lowercase letters on the bars indicate significant differences in different treatments and days, respectively (p < 0.05).

Fig. 2 a illustrates the microbiological changes of white-leg shrimp with different treatments during ice storage. TVC of all samples increased over storage time. On the last day, the TVC of Ch-LHE and N-LHE treatments reached values of 3.99 and 4.08 log CFU/g. These values are considered microbiologically acceptable for both freshwater and marine species, as they are below the threshold of 7 log CFU/g (Foods, I. C. O. M. S. F.,and Foods–ICMFS, I. C. O. M. S. F., 1986). The addition of LHE to chitosan and nanoliposome coatings resulted in improved antibacterial properties of these coatings. The significant increase in antimicrobial activity observed when the extract was loaded into nanoliposomes is likely attributed to the interaction of liposomes with bacterial cells. The mechanism of action of liposomes depends on the cell type and the physicochemical properties of the liposome membrane. Liposomes enhance cell transport and facilitate the release of active ingredients inside the cell. Pinelli et al. (2021) observed similar effects of essential oil nanoemulsions in controlling *Clostridium sporogenes* in cooked meat products (mortadella), suggesting them as alternative natural antimicrobial compounds. They suggested the use of essential oil nanoemulsions as a preservative in meat products. Additionally, previous research has demonstrated that edible coatings containing chitosan can effectively prevent microbial growth in shrimp. Homayonpour et al. (2021) found that encapsulating sardine fillets with free/nano *Cuminum cyminum* L. essential oil in chitosan coating significantly improved sensory, chemical, and qualitative properties, while also reducing bacterial population growth.

#### 3.3.2 Psychrophilic bacterial counts (PBC)

Psychrophilic bacterial counts (PBC), a significant contributor to shrimp decay during storage, remain active in the freezing medium. As the storage duration increased, PBC increased in all samples (Fig. 2b). However, samples treated with Ch-LHE exhibited a significant decrease in PBC, with a value of 4.3 log CFU/g, compared to other treatments (*p* < 0.05). Therefore, shrimp treatment with LHE, particularly when combined with chitosan coating, exhibited the ability to delay the growth of psychrophilic bacteria during ice storage. These findings suggest that chitosan coating effectively limits bacterial activity in shrimp. Essentially, the synergistic effect of chitosan coatings with strong antimicrobial properties is enhanced when combined with LHE. The use of chitosan coating or plant extracts to enhance shrimp quality has been documented in previous research. For instance, Nirmal and Benjakul (2012) found that the application of green tea extract (0.1%) reduced PBC in Pacific white shrimp during cold storage for 10 days. Alparslan and Baygar (2017) also concluded that the use of orange peel essential oil prevented the increase of PBC in shrimps compared to the control group. In another study, edible chitosan film containing encapsulated garlic essential oil effectively reduced the growth of spoilage bacteria in vacuum-packed sausages for 50 days compared to a control sample (Esmaeili et al., 2020).

### 3.4 Texture profile analysis

**Fig. 3:**
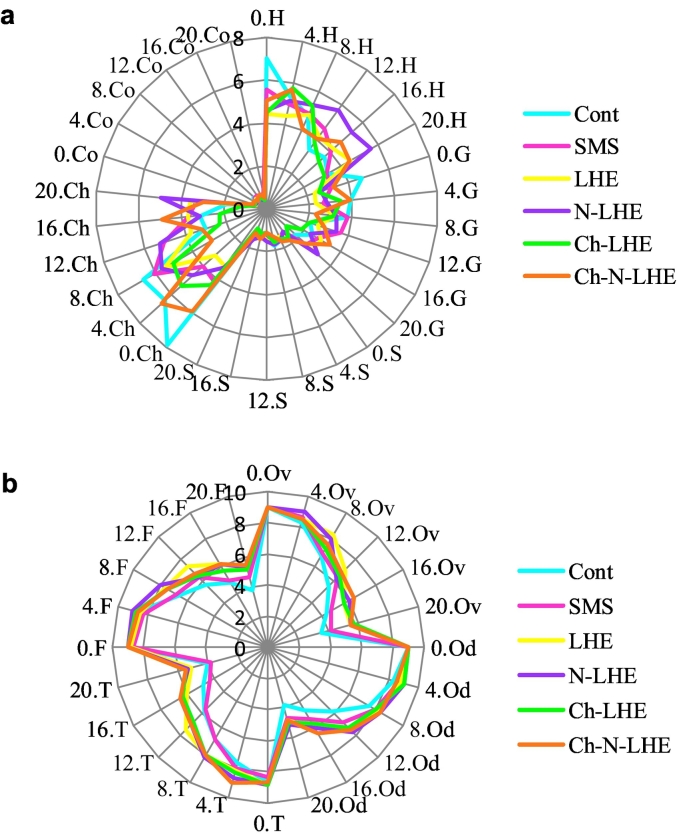
Texture changes (a) and sensory score (b) (mean) of white leg shrimp with various treatments during ice storage. H = Hardness (N), G = Gumminess (N), S = Springiness (mm), Ch = Chewiness (N mm), Co = Cohesiveness. Days = 0, 4, 8, 12, 16, 18, 20. Ov = Overall acceptability, Od = Odor, T = Texture, F = Flavor.

For seafood, texture characteristics are a significant quality criterion. Fig. 3 a presents the average values of tissue properties of shrimp samples during storage. The force required to crush the specimen, measured as hardness, represents a mechanical texture property (Liu et al., 2022). The results indicated a significant decrease in shrimp hardness values with storage time for most samples, although LHE treatment did not exhibit any significant changes (p < 0.05). These findings align with those of Sun et al. (2023), who reported that the hardness of shrimp samples initially increased and then decreased with increasing magnetic field intensity. Gumminess, characterized by low hardness but high cohesion, is often found in semi-solid products (Casas, Martinez, Guillen, Pin, & Salmeron, 2006). The gumminess of shrimp showed significant reduction in some treatments during ice storage. However, both LHE and N-LHE treatments demonstrated significant improvement compared to the control and SMS groups throughout storage. Springiness, a mechanical tissue property associated with the speed and degree of recovery from a deforming force Wang, Lei, et al. (2018), was observed to increase significantly with storage time in the N-LHE treatment. In a study by Gokoglu et al. (2022), it was found that adding an anti-browning agent to ice exhibited a greater protective effect on the tissue properties, including springiness and gumminess, of giant red shrimp compared to the immersion method. Chewiness, characterized by the feeling of tightness in the mouth due to the springiness and gumminess of food, resulting in long-term elastic resistance (Casas et al., 2006), decreased on the last day of storage in most treatments except LHE and N-LHE (Fig. 3a). The lower levels of chewiness indicate muscle softening during processing. Cohesiveness, a feature of biomechanical tissue representing the rate at which food deforms before breaking, exhibited significant increases in treatments LHE and SMS, while no change was observed in N-LHE-treated samples. Variations in cohesiveness values may stem from alterations in the internal muscle bonds of shrimp during processing. These changes in shrimp tissue during storage are attributed to proteolysis caused by endogenous and microbial enzymes acting on myofibrillar proteins, leading to the weakening of connective tissue (Liu et al., 2022). These findings align with those of Yuan et al. (2016), who demonstrated that shrimp coated with a combination of PPE and chitosan film could enhance shelf life and improve texture parameters of *Vannamei* shrimp.

### 3.5 Sensory evaluation

Fig. 3b illustrates the changes in sensory qualities of white-leg shrimp during a 20-day ice storage period. Evaluation of overall acceptability, odor, texture, and flavor was conducted. Initially, all samples received a rating of 9, indicating high acceptability, with no significant differences observed in liking among treatments (*p* < 0.05). During cold storage, there was a general decrease in the sensory scores of shrimp. The reduction in sensory scores was notably higher in the control and SMS treatments, with scores of 3.5 and 4.16, respectively, compared to other treatments. Shrimps with scores lower than five were deemed unsuitable due to characteristics such as rotten odor, lack of radiance, soft and inflexible texture, and lack of complete acceptance. By the 16th day of storage, the control and SMS treatments were deemed unacceptable due to the presence of unpleasant odors, while shrimp coated with different types of LHE still maintained slight acceptability by the 20th day. These results suggest a potential synergistic effect between the plant extract and the antioxidant and antimicrobial properties of chitosan. These findings underscore the effectiveness of LHE coating in preserving the sensory quality of shrimp, with the possibility of nanoliposomes enhancing the protective properties of chitosan coatings. Comparable results were observed in other studies; for instance, Zomorodian et al. (2023) found that adding *Zataria multiflora* essential oil to chitosan coating, whether in free or encapsulated emulsion form, was highly effective in preserving the smell, texture, and red color of salmon trout over 16 days of storage in the refrigerator. Similarly, *Pulicaria gnaphalodes* aqueous extract in nanoencapsulated form emerged as the most effective treatment for maintaining color and overall acceptance of rainbow trout over 14 days, with impressive scores of 4.12 and 4.36, as reported by Mehdizadeh et al. (2021). Additionally, Homayonpour et al. (2021) highlighted the superior sensory outcomes of sardine fillets treated with nanoliposomes containing free *Cuminum cyminum* L. essential oil, which exhibited the highest acceptance score and favorable taste during 16 days of refrigerated storage.

### 3.6 Color

Color evaluation serves as a crucial aspect in assessing product acceptance among consumers, providing an objective measure of quality. The recorded values of L*, a*, b*, C, H, and ΔE for the different treatments are presented in Table 1. Over the storage period, an increase in L* values was noted, accompanied by decreases in a*, b*, chroma (C), and hue angle (H) compared to fresh shrimp samples. Prior studies have associated a decrease in L* values and an increase in a* values with browning phenomena (Li et al., 2022). In this study, Ch-LHE treatment showed relatively higher values of L* and at the same time lower values of ΔE. On the final day of storage, the ΔE values recorded for Ch-LHE and Ch-N-LHE treatment shrimps, measuring 9.79 and 11.31, respectively, were notably lower compared to those of the control and SMS-treated shrimps (*p* < 0.05). ΔE serves as a comprehensive indicator of changes in L*, a*, and b* values, with higher values indicating greater color changes over time. These results echo findings from a recent study by Kamali et al. (2023), where shrimps coated with chitosan and *Ulva intestinalis* sulfated polysaccharide exhibited minimal color changes, with a ΔE value of 9.43, throughout the ice storage period. In another investigation by Wang, Lei, et al. (2018), the observed increase in ΔE values from 3 to 6 after chitosan-carvacrol packaging and 10 days of ice storage underscores the dynamic nature of color alterations in stored shrimp samples. Understanding the perceptibility threshold for ΔE values—where differences exceeding 3.0 become discernible to consumers—provides important context for interpreting color changes. Consequently, the utilization of LHE chitosan nanoliposome coating in our study likely contributes to delaying color changes in shrimp, potentially attributed to the anti-melanosis properties of glabridin found in the extract.

**Table 1:** Changes in shrimp shell color measurement (mean ± SD) of shrimp with different treatments (*n* = 6).

color measurement	days of storage						
	Treatments	0	4	8	12	16	20
L*	Cont	83.91 ± 0.46 ^Aa^	80.86 ± 0.62 ^Bb^	76.01 ± 0.6 ^Bc^	73.51 ± 0.64 ^CDd^	71 ± 0.52 ^Ce^	68.91 ± 0.62 ^Cf^
	SMS	83.1 ± 0.6 ^Ba^	82.85 ± 0.73 ^Aa^	76.91 ± 0.44 ^ABb^	73.3 ± 0.85 ^Dc^	71.13 ± 0.61 ^Cd^	68.81 ± 0.56 ^Ce^
	LHE	82.8 ± 0.54 ^BCa^	81.13 ± 0.77 ^Bb^	77.11 ± 0.62 ^Ac^	74.75 ± 0.4 ^Bd^	72.7 ± 0.51 ^Be^	70.81 ± 0.29 ^Bf^
	N-LHE	82.63 ± 0.37 ^BCa^	79.9 ± 0.43 ^Cb^	76.36 ± 1.4 ^ABc^	74.3 ± 0.68 ^BCd^	72.73 ± 0.64 ^Be^	70.83 ± 0.29 ^Bf^
	Ch-LHE	81.58 ± 0.67 ^Da^	78.65 ± 0.37 ^Db^	77.05 ± 0.38 ^ABc^	75.61 ± 0.59 ^Ad^	73.73 ± 0.7 ^Ae^	71.95 ± 0.48 ^Af^
	Ch-N-LHE	82.21 ± 0.63 ^CDa^	78.11 ± 0.72 ^Db^	76.9 ± 0.92 ^ABc^	75.08 ± 0.87 ^ABd^	72.9 ± 0.63 ^Be^	71.15 ± 0.41 ^Bf^

a*	Cont	3.5 ± 0 ^Bc^	3.5 ± 0 ^Bc^	3.76 ± 0.41 ^Bbc^	4.03 ± 0.41 ^BCb^	4.03 ± 0.41 ^Bb^	4.56 ± 0.41 ^Aa^
	SMS	3.5 ± 0 ^Bc^	3.5 ± 0 ^Bc^	3.76 ± 0.41 ^Bbc^	3.76 ± 0.41 ^Cbc^	4.03 ± 0.41 ^Bab^	4.3 ± 0 ^Aa^
	LHE	3.9 ± 0.43 ^Abc^	3.63 ± 0.32 ^Bc^	4.3 ± 0 ^Aab^	3.76 ± 0.41 ^Cc^	4.3 ± 0 ^ABab^	4.43 ± 0.6 ^Aa^
	N-LHE	3.48 ± 0.04 ^Bc^	3.5 ± 0 ^Bc^	4.56 ± 0.41 ^Aa^	4.16 ± 0.32 ^ABCb^	4.56 ± 0.41 ^Aa^	4.3 ± 0 ^Aab^
	Ch-LHE	3.63 ± 0.32 ^ABb^	3.63 ± 0.32 ^Bb^	4.56 ± 0.41 ^Aa^	4.3 ± 0 ^ABa^	4.3 ± 0 ^ABa^	4.3 ± 0 ^Aa^
	Ch-N-LHE	3.76 ± 0.41 ^ABc^	4.03 ± 0.41 ^Abc^	4.3 ± 0 ^Aab^	4.56 ± 0.41 ^Aa^	4.3 ± 0 ^ABab^	4.3 ± 0 ^Aab^

b*	Cont	7.23 ± 0.41 ^Ca^	6.96 ± 0.41 ^Ca^	6.16 ± 0.41 ^Ab^	5.36 ± 0.41 ^Bc^	5.36 ± 0.41 ^Bc^	5.63 ± 0.41 ^ABc^
	SMS	8.16 ± 0.08 ^Aa^	7.1 ± 0.43 ^BCb^	6.03 ± 0.32 ^Ac^	5.36 ± 0.41 ^Bd^	5.1 ± 0 ^Bd^	5.1 ± 0 ^Cd^
	LHE	8.05 ± 0.28 ^Aa^	7.36 ± 0.32 ^ABb^	6.1 ± 0.93 ^Ac^	5.9 ± 0 ^Ac^	5.9 ± 0 ^Ac^	5.9 ± 0 ^Ac^
	N-LHE	7.96 ± 0.36 ^Aa^	7.5 ± 0 ^Ab^	6.3 ± 0.43 ^Ac^	5.9 ± 0 ^Ade^	6.16 ± 0.41 ^Acd^	5.76 ± 0.32 ^Ae^
	Ch-LHE	7.5 ± 0 ^BCa^	7.5 ± 0 ^Aa^	6.3 ± 0.43 ^Ab^	5.9 ± 0 ^Ac^	5.9 ± 0 ^Ac^	5.9 ± 0 ^Ac^
	Ch-N-LHE	7.61 ± 0.28 ^Ba^	7.5 ± 0 ^Aa^	6.03 ± 0.32 ^Ab^	6.16 ± 0.41 ^Ab^	5.9 ± 0 ^Ab^	5.36 ± 0.41 ^BCc^

C	Cont	8.03 ± 0.37 ^Ca^	7.79 ± 0.37 ^Ca^	7.23 ± 0.31 ^Bb^	6.71 ± 0.5 ^CDc^	6.71 ± 0.5 ^Bc^	7.27 ± 0.04 ^Ab^
	SMS	8.88 ± 0.07 ^Aa^	7.91 ± 0.39 ^BCb^	7.12 ± 0.3 ^Bc^	6.56 ± 0.46 ^Dd^	6.61 ± 0.41 ^Bd^	6.67 ± 0 ^Bd^
	LHE	8.95 ± 0.39 ^Aa^	8.21 ± 0.35 ^ABb^	7.48 ± 0.74 ^ABc^	7 ± 0.22 ^BCc^	7.3 ± 0 ^Ac^	7.39 ± 0.35 ^Ac^
	N-LHE	8.69 ± 0.34 ^ABa^	8.27 ± 0 ^Ab^	7.79 ± 0.43 ^Ac^	7.22 ± 0.17 ^Bd^	7.68 ± 0.3 ^Ac^	7.19 ± 0.25 ^Ad^
	Ch-LHE	8.33 ± 0.15 ^BCa^	8.33 ± 0.15 ^Aa^	7.79 ± 0.25 ^Ab^	7.3 ± 0 ^Bc^	7.3 ± 0 ^Ac^	7.3 ± 0 ^Ac^
	Ch-N-LHE	8.5 ± 0.27 ^Ba^	8.52 ± 0.19 ^Aa^	7.41 ± 0.26 ^ABb^	7.68 ± 0.3 ^Ab^	7.52 ± 0.34 ^Ab^	6.88 ± 0.32 ^Bc^

H	Cont	1.11 ± 0.02 ^BCa^	1.1 ± 0.02 ^ABa^	1.02 ± 0.06 ^Ab^	0.92 ± 0.04 ^Bc^	0.92 ± 0.04 ^Ac^	0.88 ± 0.08 ^ABc^
	SMS	1.16 ± 0 ^Aa^	1.11 ± 0.02 ^ABb^	1.01 ± 0.05 ^Ac^	0.95 ± 0.05 ^ABd^	0.9 ± 0.05 ^Ae^	0.87 ± 0 ^Be^
	LHE	1.12 ± 0.03 ^BCa^	1.11 ± 0.03 ^ABa^	0.95 ± 0.07 ^Abc^	1 ± 0.04 ^Ab^	0.94 ± 0 ^Abc^	0.92 ± 0.06 ^ABc^
	N-LHE	1.15 ± 0.01 ^ABa^	1.13 ± 0 ^Aa^	0.94 ± 0.05 ^Ab^	0.95 ± 0.03 ^ABb^	0.93 ± 0.06 ^Ab^	0.92 ± 0.02 ^ABb^
	Ch-LHE	1.12 ± 0.03 ^BCa^	1.12 ± 0.03 ^Aa^	0.94 ± 0.06 ^Ab^	0.94 ± 0 ^Bb^	0.94 ± 0 ^Ab^	0.94 ± 0 ^Ab^
	Ch-N-LHE	1.11 ± 0.04 ^Ca^	1.07 ± 0.04 ^Ba^	0.95 ± 0.02 ^Ab^	0.93 ± 0.06 ^Bbc^	0.94 ± 0 ^Abc^	0.89 ± 0.03 ^ABc^

ΔE	Cont	–	3.15 ± 0.41 ^Be^	8.01 ± 0.64 ^Ad^	10.61 ± 0.57 ^Ac^	13.07 ± 0.45 ^Ab^	15.12 ± 0.48 ^Aa^
	SMS	–	1.31 ± 0.4 ^De^	6.56 ± 0.64 ^Bd^	10.21 ± 0.83 ^Ac^	12.37 ± 0.57 ^Bb^	14.63 ± 0.39 ^Aa^
	LHE	–	1.97 ± 0.38 ^Ce^	6.11 ± 0.65 ^BCd^	8.35 ± 0.39 ^Bc^	10.34 ± 0.3 ^Cb^	12.2 ± 0.29 ^Ba^
	N-LHE	–	2.79 ± 0.29 ^Be^	6.62 ± 1.27 ^Bd^	8.62 ± 0.57 ^Bc^	10.14 ± 0.53 ^CDb^	12.04 ± 0.25 ^Ba^
	Ch-LHE	–	2.93 ± 0.58 ^Be^	4.82 ± 0.42 ^Dd^	6.22 ± 0.31 ^Dc^	8.04 ± 0.24 ^Eb^	9.79 ± 0.39 ^Da^
	Ch-N-LHE	–	4.13 ± 0.62 ^Ae^	5.59 ± 0.73 ^CDd^	7.36 ± 0.88 ^Cc^	9.49 ± 0.96 ^Db^	11.31 ± 0.82 ^Ca^

Cont: Control; SMS: Sodium Metabisulphite; LHE: LHE coating; N-LHE: LHE Nanoliposome; Ch-LHE: LHE Chitosan coating; Ch-N-LHE: LHE Nanoliposome Chitosan coating.

Uppercase and lowercase letters indicate significant differences in different treatments and days, respectively (*p* < 0.05).

**Table 2:** Changes in physical and biochemical analyses (mean ± SD) of shrimp with different treatments (*n* = 3).

Physical and Biochemical analyses	days of storage						
	Treatments	0	4	8	12	16	20
Weight loss (%)	Cont	–	27.76 ± 0.88 ^Bd^	30.35 ± 0.96 ^Bc^	31.83 ± 1.01 ^Bbc^	33.53 ± 0.96 ^Bb^	36.19 ± 0.94 ^Ba^
	SMS	–	34.99 ± 1.05 ^Ac^	38.78 ± 1.27 ^Ab^	41.06 ± 1.33 ^Aab^	42.57 ± 1.26 ^Aa^	43.29 ± 1.33 ^Aa^
	LHE	–	15.96 ± 0.21 ^Dd^	27.85 ± 0.5 ^Cc^	32.01 ± 0.03 ^Bb^	32.38 ± 0.08 ^BCb^	35.02 ± 0.21 ^Ba^
	N-LHE	–	6.74 ± 0.22 ^Ec^	10.56 ± 0.25 ^Eb^	10.87 ± 0.32 ^Eb^	13.62 ± 0.39 ^Ea^	13.83 ± 0.33 ^Da^
	Ch-LHE	–	24.4 ± 0.46 ^Cc^	28.33 ± 0.49 ^BCb^	29.39 ± 0.45 ^Cab^	30.23 ± 0.52 ^Ca^	30.49 ± 0.58 ^Ca^
	Ch-N-LHE	–	22.04 ± 1.97 ^Cb^	24.78 ± 1.39 ^Db^	25.05 ± 1.36 ^Db^	25.67 ± 1.42 ^Db^	32.2 ± 1.95 ^Ca^

pH	Cont	6.7 ± 0.01 ^Ae^	6.81 ± 0.01 ^Ad^	6.78 ± 0.01 ^Dd^	6.9 ± 0.01 ^CDc^	6.99 ± 0.01 ^Eb^	7.57 ± 0 ^Aa^
	SMS	6.57 ± 0.02 ^Be^	6.78 ± 0 ^Bd^	6.86 ± 0 ^Cc^	6.86 ± 0.02 ^Dc^	7.15 ± 0.01 ^Db^	7.53 ± 0.04 ^Aa^
	LHE	6.48 ± 0.01 ^Ce^	6.63 ± 0 ^Ed^	6.8 ± 0.01 ^Dc^	6.95 ± 0.07 ^Cb^	7.37 ± 0.02 ^Ba^	7.37 ± 0.03 ^BCa^
	N-LHE	6.44 ± 0.01 ^De^	6.64 ± 0 ^Ed^	7.27 ± 0.04 ^Bb^	7.05 ± 0.01 ^Bc^	7.45 ± 0.02 ^Aa^	7.43 ± 0.02 ^Ba^
	Ch-LHE	6.41 ± 0 ^Dd^	6.75 ± 0 ^Cc^	7.27 ± 0.01 ^Bb^	7.38 ± 0.01 ^Aa^	7.28 ± 0 ^Cb^	7.24 ± 0.05 ^Db^
	Ch-N-LHE	6.43 ± 0.02 ^Dd^	6.7 ± 0.01 ^Dc^	7.49 ± 0.01 ^Aa^	7.45 ± 0 ^Aa^	7.28 ± 0.01 ^Cb^	7.31 ± 0.01 ^CDb^

Cook loss (%)	Cont	28.13 ± 0.08 ^Bc^	26.06 ± 0.45 ^Bd^	27.6 ± 0.15 ^Cc^	34.6 ± 0.86 ^Aa^	33.37 ± 0.64 ^Db^	14.62 ± 0.14 ^De^
	SMS	24.11 ± 0.08 ^Ca^	23.75 ± 1.17 ^CDa^	19.51 ± 0.01 ^Db^	24.12 ± 0.45 ^Da^	20.34 ± 0.31 ^Fb^	16.03 ± 0.42 ^Cc^
	LHE	32.48 ± 0.76 ^Ab^	26.72 ± 1.23 ^Bd^	37.87 ± 1.04 ^Aa^	30.04 ± 0.17 ^Bc^	37.87 ± 0.7 ^Aa^	18.24 ± 0.23 ^Be^
	N-LHE	27.83 ± 0.27 ^Bb^	24.65 ± 0.82 ^BCd^	37.5 ± 0.32 ^Aa^	26.16 ± 0.96 ^Cc^	36.42 ± 0.44 ^Ba^	24.15 ± 0.38 ^Ad^
	Ch-LHE	28.82 ± 1.29 ^Bab^	22.25 ± 0.25 ^Dc^	27.89 ± 0.61 ^Cb^	30.42 ± 1.1 ^Ba^	30.15 ± 0.55 ^Ea^	17.97 ± 0.1 ^Bd^
	Ch-N-LHE	33.7 ± 0.18 ^Aab^	32.44 ± 0.59 ^Abc^	31.78 ± 1.1 ^Bc^	31.09 ± 0.09 ^Bc^	34.78 ± 0.58 ^Ca^	15.06 ± 0.35 ^Dd^

WHC (%)	Cont	83.7 ± 0.31 ^Be^	88.27 ± 0.25 ^Cb^	85 ± 0.03 ^Cd^	91.76 ± 0.01 ^Aa^	87.27 ± 0.02 ^Cc^	80.97 ± 0.36 ^Df^
	SMS	86.15 ± 0.8 ^Ac^	89.28 ± 0.71 ^BCb^	86.17 ± 0.54 ^BCc^	91.83 ± 0.45 ^Aa^	86.24 ± 0.87 ^Cc^	88.52 ± 1.65 ^Abc^
	LHE	86.06 ± 0.16 ^Ab^	86.73 ± 0.5 ^Db^	83.56 ± 0.72 ^Dc^	91.88 ± 0.63 ^Aa^	83.59 ± 0.72 ^Dc^	81 ± 0.07 ^Dd^
	N-LHE	84.65 ± 0.42 ^ABd^	93.91 ± 0.41 ^Aa^	86.05 ± 0.28 ^BCc^	88.48 ± 0.03 ^Cb^	87.67 ± 0.84 ^BCb^	83.58 ± 0.81 ^Cd^
	Ch-LHE	83.01 ± 1.08 ^Bc^	89.97 ± 0.32 ^Bb^	89.29 ± 0.55 ^Ab^	92.18 ± 0.39 ^Aa^	89.75 ± 0.39 ^Ab^	80.48 ± 0.06 ^Dd^
	Ch-N-LHE	70.79 ± 0.89 ^Cc^	89.01 ± 0.45 ^BCa^	86.33 ± 0.51 ^Bb^	89.76 ± 0.41 ^Ba^	88.88 ± 0.32 ^ABa^	86.12 ± 1.05 ^Bb^

Cont: Control; SMS: Sodium Metabisulphite; LHE: LHE coating; N-LHE: LHE Nanoliposome; Ch-LHE: LHE Chitosan coating; Ch-N-LHE: LHE Nanoliposome Chitosan coating.

Uppercase and lowercase letters indicate significant differences in different treatments and days, respectively (*p* < 0.05).

### 3.7 Evaluation of shrimp browning using two instrumental and objective methods

**Fig. 4:**
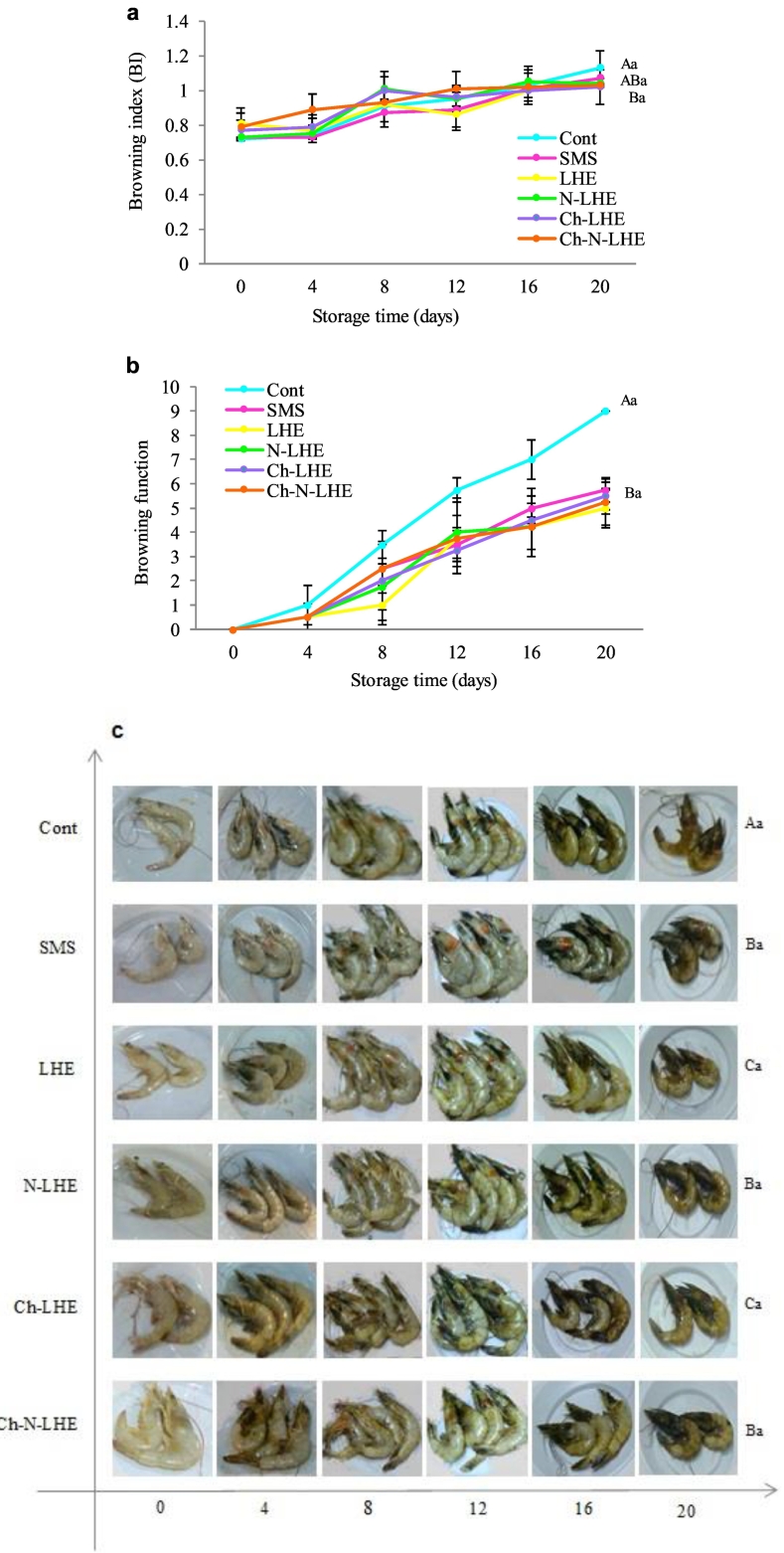
Changes in Browning index (a), Browning function (b) (mean ± SD), and Expansions of browning (c) of white leg shrimp with various treatments during ice storage. Uppercase and lowercase letters on the bars indicate significant differences in different treatments and days, respectively (p < 0.05), and the bars indicate the standard deviation (n = 3).

Browning processes impact nutritional quality and appearance, diminishing consumer acceptability and imposing significant economic burdens on seafood processing industries. The browning index (BI), browning function, and appearance of raw white-leg shrimp are depicted in Fig. 4a, b, and c. Shrimp browning, as assessed by both instrumental (BI) and visual measurement methods (browning function), increased progressively over the 20-day storage period, signifying the occurrence of the Maillard reaction. In summary, it can be speculated that the Maillard reaction may directly contribute to the color deterioration of shrimp during storage. This reaction is closely linked to pigment formation in crustaceans, with melanin, a brown nitrogen-containing polymer, being the end product. The quantity of melanin produced is quantified by the browning index (BI). In this study, treatments with different LHE coatings exhibited relatively lower browning values compared to the control during storage. Specifically, the Ch-LHE (1.02 and 5.5) and LHE (1.02 and 5) treatments demonstrated the most effective inhibition of shrimp browning. However, lack of significant difference in browning function between treatments containing LHE and SMS at the end of storage (*p* < 0.05) indicates that the results of the instrumental measurements (BI) align with the visual perception results of the panel group evaluation (browning function). Studies have demonstrated that licorice contains inhibitory compounds with wide-ranging applications. Glabridine, extracted from licorice, is a potent inhibitor of tyrosinase owing to its high antioxidant activity. Licorice extract has been demonstrated to inhibit fungal tyrosinase and melanin production in cells under laboratory conditions (Nerya et al., 2003). Additionally, chitosan's delay in the appearance of black spots in shrimp can be attributed to its chelating function and oxygen removal from the coating, which subsequently reduces polyphenol oxidase activity. The findings align with previous research, such as a study where shrimps coated with chitosan solution containing 10% and 20% polyethylene exhibited reduced development of dark pigment during 9 days of storage (Isfran et al., 2023). This effect can be attributed to the presence of phenolic compounds in the chitosan coating, which mitigate lipid oxidation in shrimp during storage. Additionally, in a recent investigation, shrimps treated with phytic acid and lactic acid and then cooked displayed the most effective inhibition of increasing browning index during 18 days of storage at 37 °C (Li et al., 2022). Consequently, glabridine in LHE, known for its potent anti-melanosis properties, when combined with chitosan and nanoliposomes, can effectively reduce the browning function of shrimp during ice storage.

## 4 Conclusion

The encapsulated or chitosan-coated licorice extract demonstrates significant efficacy in inhibiting lipid oxidation, microbial count, weight loss, and cooking loss of shrimp compared to control and sodium metabisulfite treatment. Moreover, it improves texture stability, sensory properties, and shrimp color. The research findings suggest that the inhibitory effect of shrimp browning with licorice root extract, with different coatings, is comparable to that of sodium metabisulfite. Therefore, it is recommended to utilize this natural herbal extract in the form of chitosan-coated nanoliposome (Ch-N-LHE) as a viable alternative to sodium metabisulfite in shrimp processing industries. However, it's worth noting that the pure form of the extract (LHE) is more cost-effective. Additionally, the potential of glabridin (the pure substance and licorice antityrosinase) in influencing the shelf life and color of shrimp remains unexplored and warrants investigation in future research endeavors.

## CRediT authorship contribution statement

**Masume Kamali:** Writing – original draft, Software, Project administration, Investigation, Data curation, Conceptualization. **Bahare Shabanpour:** Validation, Supervision, Methodology, Formal analysis, Data curation, Conceptualization. **Parastoo Pourashouri:** Writing – review & editing, Visualization, Project administration, Methodology, Investigation, Data curation. **Moazameh Kordjazi:** Writing – review & editing, Resources, Investigation.

## Declaration of competing interest

The authors declare that they have no known competing financial interests or personal relationships that could have appeared to influence the work reported in this paper. The authors declare that they have no conflict of interest.

## Data Availability

The authors are unable or have chosen not to specify which data has been used.
